# Active and healthy ageing in urban environments: laying the groundwork for solution-building through citizen science

**DOI:** 10.1093/heapro/daac126

**Published:** 2022-09-14

**Authors:** Grace E R Wood, Jessica Pykett, Afroditi Stathi

**Affiliations:** School of Sport, Exercise and Rehabilitation Sciences, University of Birmingham, Edgbaston Park Road, Birmingham, West Midlands, B15 2TT, UK; School of Geography, Earth and Environmental Sciences, University of Birmingham, Ring Road S, Birmingham, West Midlands, B15 2TT, UK; School of Sport, Exercise and Rehabilitation Sciences, University of Birmingham, Edgbaston Park Road, Birmingham, West Midlands, B15 2TT, UK

**Keywords:** older adults, citizen social science, age-friendly, urban health, well-being

## Abstract

Urban age-friendly initiatives strive to promote active and healthy ageing by addressing urban influences that impact individuals as they age. Collaborative community partnerships with multi-level stakeholders are crucial for fostering age-friendly initiatives that can transform urban community health. Employing a citizen social science (CSS) approach, this study aimed to engage older adults and stakeholders in Birmingham, UK, to (i) identify key urban barriers and facilitators to active and healthy ageing, and (ii) facilitate collaboration and knowledge production to lay the groundwork for a citizen science project. Older adults (*n* = 16; mean age = 72(7.5 SD); 11 female) and community stakeholders (*n* = 11; 7 female) were engaged in six online group discussions, with audio recordings transcribed and thematically analysed to present key urban barrier and facilitator themes. Ageism, winter, technology and safety were barriers identified by both groups. Outdoor spaces and infrastructure, transportation, community facilities, and Covid-19 pandemic were identified as barriers and/or facilitators. Older adults identified the ageing process as a barrier and diversity of the city, health and mobility and technology as facilitators. For stakeholders, barriers were deprivation and poverty, gender differences, and ethnicity, whereas age-inclusive activities were a facilitator. Organic and active opportunities for older adults and stakeholders to connect, co-produce knowledge on urban environments and share resources presented foundations of solution-building and future collaboration. CSS effectively facilitated a range of stakeholders across local urban spaces to collaborate and co-produce ideas and solutions for enhancing local urban environments to promote active and healthy ageing.

## INTRODUCTION

The idea of age-friendly cities has become a global movement that considers the role urban environments present for age-related challenges (Davern *et al.*, 2020). Urban age-friendly initiatives strive to promote active and healthy ageing by addressing urban influences that impact individuals as they age (van Hoof *et al.*, 2018; World Health Organization, 2018). Considering age-friendly elements across the environment, services, structures and policies, the age-friendly city can promote active ageing and quality of life by optimizing opportunities for health, participation and security in specific places (World Health Organization, 2007).

Employing a bottom-up participatory approach, the age-friendly agenda brings together the interests from older adults and urban planners to collaborate, co-produce knowledge and foster age-friendly spaces. Yet developing and actualizing inclusive and supportive age-friendly environments is argued to present a key gap for governance and public policy (Buffel and Phillipson, 2016; Murtagh *et al.*, 2021). Studies engaging older residents to identify urban influences of active and healthy ageing highlighted that engagement of residents during preliminary design and planning stages was missing (James and Buffel, 2022; Wood *et al.*, 2022). This strongly suggests a missed opportunity to take a place-based and community-engaged approach to addressing the health inequalities experienced between different age groups, such as differences in physical mobility attributed to age-related impairments and physical limitations (Rosso *et al.*, 2011; Bowering, 2019). A lack of responsibility, intent and understanding from local and regional governments, alongside challenges of translating empowerment practices, are also undermining participatory processes and their beneficial health outcomes (Steels, 2015; Joy, 2018; Popay *et al.*, 2020). Without collaborative governance across all levels, a lack of understanding and municipal capacity will be present in age-friendly initiatives (Jackisch *et al.*, 2015; Russell *et al.*, 2022).

Collaborative governance and participatory initiatives have become increasingly present in the planning domain, developing public–private partnerships throughout planning processes (Ghose, 2005). However, the planning domain is identified as lacking opportunities and processes for local community members to participate *and* inform policy (Parker and Murray, 2012). An example includes Neighbourhood Planning (UK Public General Acts, 2011) which aimed to employ participatory processes to facilitate local-level decision-making in the UK (Parker and Murray, 2012; Brookfield, 2016). This approach was perceived as tokenistic in its collaborative practices to give power to the community, containing limitations in its democratic inquiry and the planning choices actually available to the local level (McGuiness and Ludwig, 2017). Power in this process was also viewed as highly unequal, with developers and planning experts negatively perceived to have a strong influence over plans due to being ‘experts’ (Brown and Chin, 2013; Field and Layard, 2017). For bottom-up and participatory approaches to be effective in the planning domain, a form of localism is required that prioritizes interactions between citizens and key actors that can act “upon the connections of power to bring actors into different conjunctions” (Bradley and Brownhill, 2017, p 253).

As the central role of urban planning is now recognized in health promotion, prevention and addressing health inequalities (Ramirez-Rubio *et al.*, 2019; Boyko *et al.*, 2021), developing coordinated and collaborative community partnerships that can bring together and empower actors from multi-level domains is crucial for connecting localized needs with local governance (Rydin, 2007; Russell *et al.*, 2022). Current initiatives aimed to empower communities are described by Popay (Popay *et al.*, 2020) to have an overpowering inward gaze, focusing on conditions or changes related to the psychosocial, neighbourhood and everyday elements. This inward gaze overlooks the outward gaze of the wider social and political changes required for health equity, reducing the ability of communities to address health concerns as a collective. In turn, there is a need for urban health promotion to further engage in community participatory, organizing and empowerment strategies that can enable shared decision-making amongst all social actors within specific governance contexts to facilitate health equity further (Orr, 2007; Popay *et al.*, 2020).

To address this, shifting away from current traditional processes of connecting communities may be required to develop connections and changes that can transform community health (Durie and Wyatt, 2013). Citizen science (CS) actively engages the public in scientific research processes, via contributory, collaborative or co-created production levels of engagement. This engagement can develop new knowledge and outcomes that drive environmental change and community transformation (King *et al.*, 2016; Rubio *et al.*, 2020; Shulla *et al.*, 2020), alongside informing public health policy about the needs and concerns of local residents (Den Broeder *et al.*, 2016). Citizen social science (CSS) incorporates community-driven CS engagement, but centres on partnerships and collective generation of knowledge from a wide range of social actors, including local residents, community members and local authorities, to position a project around social concerns. This includes building on the engagement and empowerment of community members achieved through community-driven CS (King *et al.*, 2019; Okop *et al.*, 2021), whilst aiming to increase the understanding of societal processes and enabling citizens to raise and reflect on social issues (Albert, 2021; Fischer *et al.*, 2021). To facilitate enhanced engagement of social actors, capacity-building methods are crucial and require collaboration from a range of individuals and organizations with differing expertise (Richter *et al.*, 2018). Overall, CSS has been employed by this study to effectively achieve a collaboration that can empower residents, providers and organizations to collaborate, forming new relationships and developing sustainable networks (Albert, 2021; de Sherbinin *et al.*, 2021; Kendall *et al.*, 2022).

The forming of these networks and collaboration across social actors will then be utilized to provide insights on bringing together a range of older adults and stakeholders to collaborate and generate knowledge on urban spaces, which can lay the groundwork for a CS project. Employing a CSS approach, this study aimed to engage older adults and stakeholders to (i) identify key urban barriers and facilitators to active and healthy ageing in local urban areas of Birmingham, UK; and (ii) to build on CSS to facilitate collaboration and knowledge production in order to form the foundations of a network that can further purpose collective policy recommendations to promote an age-friendly society.

## METHODS

### Context

The ‘Improving Your Local Area’ CS project aimed to (i) employ the *Our Voice* CS for health equity approach (King *et al.*, 2019) to engage older adults and multi-level stakeholders, and (ii) co-create urban recommendations for promoting active and healthy ageing in the city of Birmingham, UK. As part of this CS project, this study provided a preliminary stage to lay the groundwork for the generation of local urban knowledge and the development of a network between older adults, stakeholders and the researcher. Utilizing a CSS at this preliminary stage, to form collaboration and new relationships across older adults and community stakeholders (Albert, 2021; de Sherbinin *et al.*, 2021) will feed into the *Our Voice* CS approach and guide the subsequent project stages based on the views and knowledge shared directly by these individuals. This is important for centring the project and its agenda setting at each stage on the needs and experiences of these individuals in their local areas (Albert *et al.*, 2021).

### Qualitative citizen social science approach

A qualitative CSS approach was employed to actively engage older adults and community stakeholders to identify local urban barriers and facilitators and build the foundations of a network that can reflect on social concerns influencing the wider ageing urban community. The approach was deemed suitable as it employs a co-production level of CS, where individuals engage throughout all stages of a project, which can facilitate the generation of new knowledge from the individual and a collective group of individuals engaged (Bonney *et al.*, 2009; King *et al.*, 2019; Fischer *et al.*, 2021). Employing CSS enables individual voices to change and new perspectives to be generated, with conflict navigated, so that ‘citizen voice’ is more than the sum of individual citizens’ voices. This can centre new knowledge around broader social aspects and encompass the expectations of a range of social actors, facilitating a broader set of outcomes for society and social sciences (Albert *et al.*, 2021).

Qualitative discussion groups called ‘Discover Together Groups’ were created. Discussion groups engaged older adults to openly identify, discuss and co-create knowledge around urban influences whilst connecting through shared and valued experiences in Birmingham (Payne and Payne, 2004; Lune and Berg, 2017). Discussion groups have been identified to facilitate essential design and construction processes with multiple stakeholders, as well as stakeholder analysis for understanding stakeholder needs, in age-friendly projects (Xiang *et al.*, 2020). Separate discussion groups following the same format were conducted for community stakeholders. Due to the Covid-19 global pandemic, the original in-person discussion groups were shifted to Zoom Video Communications (5.8.4 (2421)).

### Citizen recruitment and location

Older adults aged 60 and above were recruited across the city of Birmingham. Recruitment material and Covid-19 study amendments were shared via email through local community organizations, partnerships and services across the whole of Birmingham. Convenience and snowball sampling was employed to recruit older adults. Individuals were telephone screened by GW to confirm demographic information, their length of residence in Birmingham and their ability to walk at least 20 minutes outside. Each individual was given information about the study, including risk assessment and ethical approval, and provided written informed consent prior to study commencement. Convenience and snowball sampling was also employed to recruit community stakeholders in urban planning and ageing-well services, who were contacted directly via email. Community stakeholders took part in separate discussion groups only and provided verbal consent. Ethical approval was received prior to study commencement.

Birmingham is the second largest city in the UK and has an estimated population of 1,141,400. Older adults aged 65 and above represent 13.1% of this population, which is expected to greatly increase to 29.5% by 2038. Birmingham is a superdiverse city with White, Asian, Black and other ethnicities present. It is also the third most deprived of the UK’s core cities, which refers to the 11 largest UK cities excluding London (OECD, 2020). Deprivation is attributed to increasing years in poor health for older adults residing in the city (Birmingham City Council, 2018). Birmingham land use is mainly urban with a widespread road network but has a significant number of green spaces covering a total of 3200 hectares (Birmingham City Council, 2006, 2021).

### Data collection

Sixteen older adults (age range = 60–87, female = 11) and 11 community stakeholders (female = 7) took part in 6 online discussion groups (60–90 minutes) exploring key urban barriers and facilitators to active and healthy ageing. The online discussion groups, which were held via Zoom Video Communications (5.8.4 (2421)), were separate for older adults only (*n* = 4 discussion groups) or community stakeholders only (*n* = 2 discussion groups) and were audio recorded by GW. One older adult discussion group included a community stakeholder from the same local area. The format of the groups included a study introduction and the use of three open-ended questions to facilitate discussions. These were shared at the start of the discussion, a strategy that has shown to facilitate stakeholder engagement and partnerships in the age-friendly agenda (Garon *et al.*, 2014). The three questions included the following:

#### Older adult group questions

- Are there any barriers in your local area that impact you from being active or healthy?
- Are there any facilitators in your local area that encourage you to be active or healthy?
- In your opinion, what could be changed in your local area to provide the opportunity to be active and healthy?

#### Community stakeholder group questions

- Are there any barriers that may impact or prevent older adults from being active and healthy?
- Are there any facilitators that may impact or allow older adults to be active and healthy?
- In your opinion, what could be changed in urban areas of Birmingham to make an impact on older adults?

### Data analysis and member checking

Discussion group audio recordings were transcribed by GW and shared with older adults and community stakeholders for member checking to confirm their accuracy. A thematic analysis was completed on the audio transcripts using NVivo 12 Software (QSR International, Australia). GW completed an inductive analysis (Braun and Clarke, 2006; Saldaña, 2016) using a mixture of semantic and latent coding (Terry *et al.*, 2017) to identify barriers and facilitator themes that emerged from the transcripts. Data coding and analysis were conducted in the following stages:

#### Stage 1: Familiarization and raw coding

GW transcribed each audio transcript to become familiar with the data. GW generated raw codes by identifying and coding all segments of a transcript to identify barriers or facilitator codes present. Both latent and semantic coding were completed based on the explicit content of what an individual said whilst applying an interpretative view to capture the meaning (Supplementary Material 1).

#### Stage 2: Constructing themes from codes

GW examined each code to combine or collapse them together to produce more meaningful codes. Themes were then developed based on the combined codes that underpin each theme present for barriers or facilitators.

#### Stage 3: Reviewing and finalizing themes

Codes and themes were initially shared with AS and JP to be examined and discussed. This discussion guided a further stage of coding completed by GW, which involved collapsing codes further to produce more meaningful themes. The second set of themes was re-shared with AS and JP to discuss and produce a final set of themes.

#### Stage 4: Member checking and grouping of themes

The audio transcript and the final set of themes were shared with older adults and community stakeholders for transcript verification (Rose and Johnson, 2020) and member checking. Member checking was employed to engage older adults and community stakeholders to establish that the data and its interpretation were an accurate representation of the online discussion groups, which can maintain the validity and credibility of data (Creswell and Miller, 2000; Candela, 2019). Themes were shared via a diagram (Supplementary Material 1), and feedback received was used to amend themes. After this process, the themes from each discussion group were grouped together to identify the common barrier and facilitator themes across all older adults and all community stakeholder groups.

## RESULTS

### Citizen scientist and community stakeholder characteristics

The majority of older adults were White British (62%), married (68%), educated to university degree level or above (75%), and lived in Birmingham for a minimum of 30 years (68%). The residences of the older adults covered 11 of the 69 wards in Birmingham. The 11 wards represented the more deprived (9%), mid ranking (18%), least deprived (55%) and affluent areas (18%) across Birmingham. Ethnic groups across the wards included White British or White other (54.7%–87.8%), Asian (3.8%–30.9%), Black (1.2%–13.2%) and other ethnicities (1%–3.2%). Two of the 11 wards (18.9%) represented similar ethnic groups to Birmingham as a city, with 1 ward (9.1%) having higher Asian ethnicities and 8 wards (72%) having higher white ethnicities (Birmingham City Council, 2022). The majority of community stakeholders were female (64%) and were from urban planning or ageing-well roles across community organizations, partnerships and services.

### Barriers to and facilitators of active and healthy ageing

**Fig. 1:**
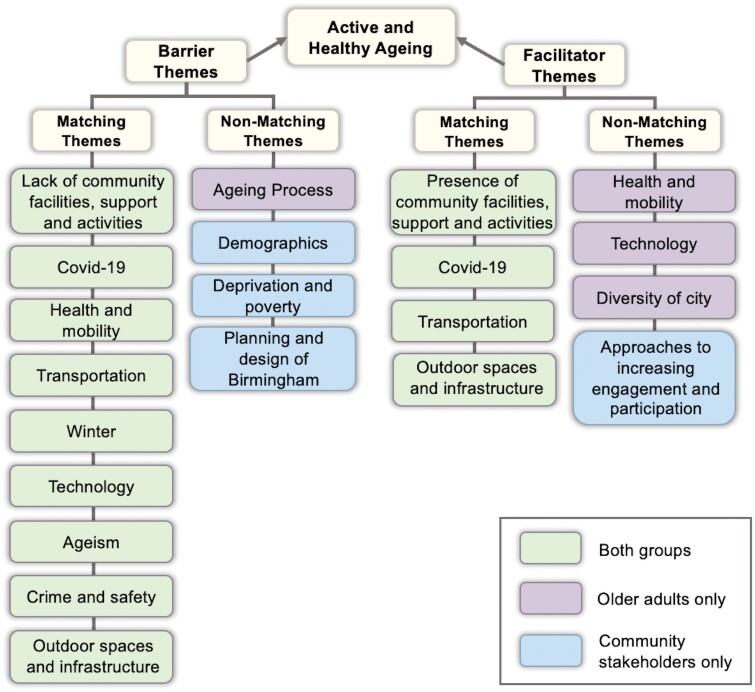
Barriers to and facilitator of active and healthy ageing themes. Themes highlighted in bold and italic are themes unique to older adults or community stakeholders and do not match between the two groups.

A total of 13 barrier themes and 8 facilitator themes were identified across the older adult and community stakeholder groups. Out of these themes, 10 barrier themes and 7 facilitator themes were identified by older adults, and 12 barrier themes and 5 facilitator themes were identified by community stakeholders (Supplementary Material 2). Overall, nine barrier themes and four facilitators matched between the two groups, and four barrier themes and four facilitator themes differed (Figure 1).

#### Matching themes between older adults and stakeholders

Nine matching barriers and four matching facilitators highlighted a range of interconnected and multi-level themes present across local urban spaces in Birmingham. The matching themes covered elements of personal (e.g. *health and mobility*, *ageism*, *crime and safety*), environmental (e.g. *outdoor spaces and infrastructure*, *transportation*, *crime and safety*), socio-cultural (e.g. *lack or presence of community support and activities*, *technology*), economic (e.g. *technology*, *transportation*, *community activities*) and political (e.g. *transportation*, *community facilities*, *technology*) domains of urban environments in Birmingham. Themes also overlapped across multiple urban domains, with the transportation theme representing movement across local urban areas (*environmenta*l), high costs of public transportation (*economic*), and the need for policy and local regulations to make public transportation more frequent and affordable (*political*). Similarly, technology was identified to facilitate online social activities and connections (*socio-cultural*), present high costs as technology advances (*economic*), and a need for digital exclusion strategies and policies to be put in place (*political*). *Covid-19 and winter* were also themes highlighted by both groups. *Covid-19* included barriers such as closed or lack of facilities for activities, decreased group activities, health concerns, and facilitators including increased outdoor activity and connecting through digital technology. *Winter* highlighted barriers such as darker evenings, reduced activity and fear of slipping due to bad weather.

#### Non-matching themes between older adults and stakeholders

Four barrier and four facilitator themes did not match between older adults and community stakeholders.

##### Older adults only

Ageing was a theme that encompassed a range of barriers when transitioning into the older adult demographic group. The ageing process was identified as a barrier in terms of becoming increasingly frail, which reduced an individual’s self-confidence, and having reduced capacity to be active and mobile. Secondly, the resources and support available as individual’s age were also highlighted as a barrier. This included a lack of support for post-retirement in relation to becoming resilient and staying active within local communities, and the need to make sure available resources such as shops are within an accessible distance. Lastly, intergenerational challenges focused on barriers with younger age groups, including difficulty to engage with younger individuals and the presence of a divide and feelings of distance with younger age groups.

Health and mobility were identified as facilitators highlighting the importance of support for walking, such as walking aids, for being active and engaging in activities. Technology was also seen to encourage activity through relevant walking apps, providing information for local activities, and enabling individuals to connect with each other and neighbours through digital platforms such as WhatsApp. Lastly, the diversity of Birmingham including a variety of people, experiences, cultures and facilities across the city was perceived as making it a vibrant, lively, and interesting place to live and participate in activities.

##### Community stakeholders only

Demographics was a barrier highlighted only by community stakeholders. This included how gender differences impact engagement and movement around the city and how active ageing is also characterized by ethnic differences due to different enabling factors for being active and healthy. Older women were identified to take part and engage more in activities than men, with a need to provide more comfortable and suitable places where older men can engage. The level of deprivation, economic deprivation and poverty were also seen as a barrier for older adults living in Birmingham. The index of multiple deprivation, which covers elements such as income, health and living environment, was identified to be one of the larger predictors for physical inactivity and was seen as a key barrier. Economic deprivation and poverty were also identified to limit older adults’ access to resources, such as the cost of transportation limiting an individual’s travel. Multiple barriers were also highlighted by community stakeholders for the planning and design of Birmingham, relating to generic planning documents that are open to interpretation, lack of specificity and sense of direction, and present a gap between what is presented in policy and what is actually delivered on the ground.

Approaches to increase the engagement and participation of older adults in activities included gamification, age-inclusive activities, a local neighbourhood approach and participant-led activities. Alongside engaging individuals, efforts to target a range of age groups, local groups and facilities via competitive and fun elements and more activities were identified. Understanding such facilitators to social participation is important for developing the CSS method.

### Developing a foundation for a CS network

**Fig. 2:**
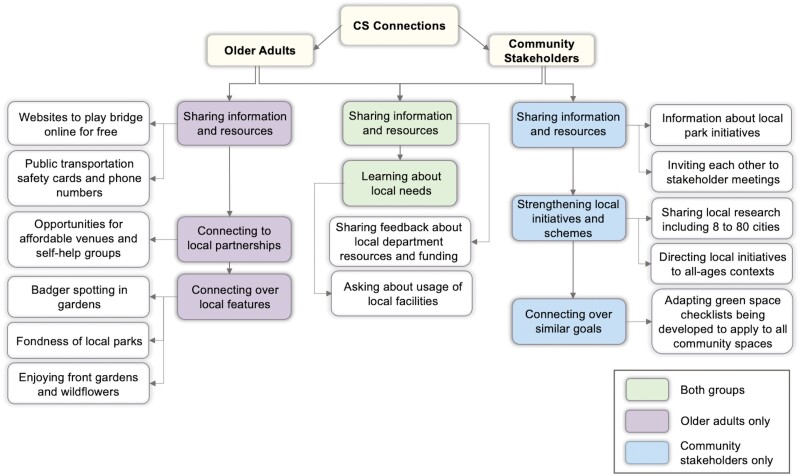
Overview of connections and sharing of resources between older adults only, community stakeholders only, and between older adults and stakeholders.

Employing CSS discussion groups at this preliminary stage facilitated organic connections and sharing of resources between older adults and between community stakeholders (Figure 2). The collaborations that occurred during each group discussion led to the sharing of information, resources and fondness of local areas, as well as solution-building.

#### Older adults

Older adults connected throughout the online discussion groups in relation to the topics being discussed, with one individual sharing websites for playing online activities such as bridge with another who could not afford in-person activities. Another shared information about a public transportation card that provides contact details for staying safe on trains. Older adults in the same local area also connected over their fondness of local parks, the presence of badgers and wildflower meadows that are being planted. Solution-building was also present, with an individual sharing details of a local community partnership for providing venues with another that was unable to afford venues to run self-help and exercise classes:

**P3 (female, 81 years)**: “I wanted a self-help group which everything was prepared, and ladies were willing to come, ladies and gents both. But I could not find, there’s no community centre or anything like that…I could not get a place”.

**P1 (female, 64 years)**: “I’m wondering whether one of the partners in the organisation I am part of is somewhere near her, there might be an opportunity …the church has a charity which would allow yoga so if I send you the information, it might be that there is somewhere near P3 that would be interested in what she’s got to offer”

#### Community stakeholders

Collaboration between community stakeholders led to the sharing of information and resources for local programmes, groups and schemes being run. Information about local park initiatives and organizations undertaking work in green spaces was shared between one group, whilst another group invited each other to local meetings happening. One group connected over their similar goals for local urban programmes, identifying ways to adapt current work in green spaces to be applicable across all community spaces. Stakeholders also shared ways to enhance each other’s schemes by sharing local research and identifying the need to put healthy ageing into an all-ages context:

**P3 (female, non-profit community organisation)**: “I think putting it in sometimes into an all-ages context might actually be more helpful in terms of moving this forward”.

**P2 (female, active travel charity)**: “On that point, have you heard of the 8 to 80 cities which is research looking at how if you make it good for eight year olds, you also make it good for a two year olds, or vice versa”.

**P1 (female, non-profit community organisation)**: “No, I haven’t but that’s exactly the point.”

## DISCUSSION

Employing a CSS approach, this study generated new knowledge about urban spaces in the context of social science. This knowledge was based on both the individual and collective voices of older adults and community stakeholders, alongside facilitating connections and resource sharing between these social actors. The main findings of this study demonstrate (i) the identification of urban barriers and facilitators to active and healthy ageing specific to the local-evel context of Birmingham; and (ii) the suitability of CSS for enabling knowledge production and collaboration at a preliminary stage.

### Urban barriers and facilitators to active and healthy ageing in local urban areas of Birmingham

Cities encompass distinct concerns at the city, community and street levels which require suitable age-friendly approaches that can address these differing needs (Phillipson, 2014; Chao, 2018). The discussion groups identified 13 barrier and 8 facilitator themes that influence active and healthy ageing in urban environments of Birmingham. These themes, which covered elements of personal, environmental, socio-cultural, economic and policy domains present in urban environments, are supported by participatory research into age-friendly environments (van Hoof *et al.*, 2019; King *et al.*, 2020), demonstrating relevance for promoting active and healthy ageing. The agreed barrier and facilitator themes amongst each discussion group and between older adults and stakeholders suggest a shared framing of local urban spaces and concerns in Birmingham (Greenfield *et al.*, 2021). This presents a promising public and private response to urban spaces that are aligned, which is crucial for sustainable age-friendly initiatives (Scharlach, 2012; Xiang *et al.*, 2020).

Considering the local context of barriers and facilitators highlights the issues and needs of older adults at the local level and can develop urban indicators that facilitate age-friendly processes in local urban environments (van Hoof *et al.*, 2021). The themes presented in this study show specificity to the local-level context of Birmingham, such as the theme *Covid-19* which encompassed a range of context-specific influences identified by both groups. The closing of churches and lesbian, gay, bisexual and transgender facilities was seen to have reduced social contact, whilst perceived increase in crime and limited capacity on buses due to health risks decreased outdoor activity. However, local WhatsApp groups increased neighbourhood connections, and socially distanced changes to cafés and shops were viewed to make spaces comfortable. Whilst it is clear that older adults have been greatly impacted across their local communities and care settings by Covid-19 (Buffel *et al.*, 2020), there is an opportunity to reconsider current processes to address the needs of older adults arising from the pandemic to further promote community health (Phillips *et al.*, 2021). For Birmingham, enhancing the public transportation system to run at full capacity and re-opening of much-needed community facilities whilst incorporating elements of social distancing may promote outdoor activity and socializing. Similarly, continuing to engage and promote technology for social activities, which will require an understanding of the specific technological needs (White *et al.*, 2020), may enable social connections to continue.

*The presence of community facilities, support and activities* are specific local facilitators to Birmingham. All groups mentioned the importance of urban allotments and open garden events for facilitating walking, gardening activities and opportunities to socialzse with other residents. Public health research evidence supports the importance of allotments for older adults, which are associated with reduced stress and better perceived social cohesion and health (Hawkins *et al.*, 2011; Soga *et al.*, 2017). Gardening activities, or horticultural therapy, also connect individuals to nature, enhance their well-being and isare beneficial for physical activity (Wang and MacMillan, 2013; Lin *et al.*, 2021). The Midlands Art Centre was another local facilitator that was noted to have promoted physical and social activities. Older adults and stakeholders would walk, cycle or take public transportation to the centre to participate in free and inclusive art and theatre activities. Engaging in these types of activities can enhance social connections, develop skills and cognitive benefits, and enrich psychological health of older adults (Yuen *et al.*, 2011; Noice *et al.*, 2014). Promoting the availability of urban allotments and art facilities that provide inclusive and free activities should be encouraged further to facilitate active and healthy ageing of older adults across Birmingham.

### CSS for facilitating collaboration and knowledge production at a preliminary stage

Employing a CSS approach at this stage facilitated discussions between older adults and stakeholders to co-produce a set of urban barriers and facilitators, which were grounded in real-world experiences and knowledge. Having a common framing of these urban influences engaged groups further to share ideas and co-produce actions in the facilitator themes. Age-inclusive sports such as cricket and engaging further with technology to address the divide between older adults and younger age groups were collectively discussed as facilitators by both groups. This indicates a sense of value in this exploratory phase in which CSS facilitated a collaborative generation of knowledge about urban spaces that surpassed consultation. Both older adults and stakeholders were able to discuss and identify collective initiatives and actions based on social concerns and embodied experiences present across urban spaces (Kythreotis *et al.*, 2019; Campos *et al.*, 2021). This provides a strong groundwork for CSS research to be driven by localized concerns of local people through directly engaging with residents and constituents and identifying ways to answer to their needs. Furthermore, it presents the foundation for strengthening collaboration and building a network of stakeholders based on their common framing of local urban spaces, demonstrating the potential for local joint action (Housley, 2018; Orton *et al.*, 2019; de Jong *et al.*, 2022).

CSS provided active opportunities to operationalize the joining of community-based knowledge and resources among older adults, stakeholders and also with the researcher. This facilitated co-production of relevant local knowledge on urban environments, rather than being solely directed by scientific practices, supporting the potential for legitimacy between these individuals for further collective and agreed-upon actions in urban spaces (Couvet *et al.*, 2008; Arnesen, 2017). Collaboration between older adults and between stakeholders through the sharing of resources and solution-building also suggests the incremental development towards the foundation of a collective network. Discovering the urban barriers for some individuals led to solution-building through shared discussions and resources. For example, older adults shared ideas for addressing unaffordable venue hire, whilst stakeholders provided suggestions for bringing their organizations together to make local initiatives stronger. The connection and generation of ideas and solutions indicate that CSS can effectively form the foundations of community-building (Freitag and Pfeffer, 2013; Greenfield *et al.*, 2021), develop future interplay between stakeholders (Albert, 2021; Campos, Monteiro and Carvalho, 2021), and facilitate a supportive online environment in which collaboration and knowledge coalition are achieved (Steels, 2015; Thomas *et al.*, 2021). Enabling connections, common actions and social cohesion can have indirect positive health impacts (Popay *et al.*, 2020) and effectively contribute towards addressing social challenges present in urban spaces (Soleri *et al.*, 2016; Thomas *et al.*, 2021).

### Strengths and areas for further research

A key strength of this study is the collaboration and shared knowledge resource facilitated by CSS. Older adults and a wide range of stakeholders collaborated to identify and discuss urban influences across Birmingham. This enabled a knowledge resource of ageing-well and urban planning processes to be shared, a collective identification of contextual and meaningful urban influences, and the foundations of a network to be formed.

A priority area for attention in future studies is the representation of voices and experiences of ethnically and gender-diverse groups of older adults. This study utilized a qualitative and highly involved approach which encompassed the voices of a small group of older adults and aimed to build a community of interest around age-friendly urban change. Participants in our study were predominantly female and White English. Females are shown to have different mobility and travel needs (Willis *et al.*, 2004; Spain, 2014) and pursue different physical and social activities in urban spaces (Pleson *et al.*, 2014; Noon and Ayalon, 2017). Birmingham is also a superdiverse city, with ethnic diversity presenting a range of different urban narratives and influences (Harries *et al.*, 2019) that require further consideration. Therefore, future research should endeavour to provide representative accounts of ethnically and gender-diverse and disadvantaged groups of older adults to further encompass and represent a wider range of voices. Further, shifting the discussion groups from in-person to online via Zoom due to the Covid-19 global excluded participants who were not digitally literate. Future research should engage with older adults both online and face to face in order to increase the representativeness of participants and outcomes.

### Future steps

CSS facilitated togetherness, networking and connection between older adults and between stakeholders to present a set of matching concerns and a sense of common purpose for urban spaces in Birmingham. These are elements that can inform decision-makers about the views of older adult residents (Den Broeder *et al.*, 2016) that can work towards improving urban community health. Utilizing the CSS research reported here, we have further engaged with older adults and community stakeholders in the next steps of our study, which goes on to employ the *Our Voice* CS for health equity framework (King *et al.*, 2019). This aims to further co-produce knowledge on urban barriers and facilitators to active and healthy ageing in Birmingham based on their voices and experiences (Thomas *et al.*, 2021). Once completed, we will evaluate and assess the CS project in relation to the strengths and weaknesses of the CS approach employed, the partnerships built throughout, and the potential possibility of future ripple effects (Welborn *et al.*, 2016).

## CONCLUSION

This study effectively engaged older adults and a range of stakeholders through a CSS approach to collaborate, share concerns and co-produce ideas for enhancing local urban environments across Birmingham. A set of urban barriers and facilitators were identified by both groups, presenting a range of matching features across personal, environmental, socio-cultural, economic and policy domains. Older adults and stakeholders collaborated during discussions to generate actions and solutions to further promote active and healthy ageing, including providing a range of age-inclusive activities. The emerging connections and resources shared between older adults and between stakeholders, alongside the alignment of matching urban barriers and facilitators, indicate the potential foundation for a collaborative network that can continue to be engaged further. Employing CSS shifted the focus of urban health promotion from targeting the individual to instead considering collaboration between individuals to identify community needs within urban environments, further supporting the increasing evidence base of the importance of place-based health promotion initiatives.

## Supplementary Material

daac126_suppl_Supplementary_Material_1

daac126_suppl_Supplementary_Material_2

## Data Availability

The data that support the findings of this study are available in Supplementary Material and from the corresponding author upon reasonable request.
