# Supplementary material for: Active and healthy ageing in urban environments: laying the groundwork for solution-building through citizen science
Source: Health Promot Int. 2022 Sep 14;37(4):daac126. doi: 10.1093/heapro/daac126 (PMC9472256; doi:10.1093/heapro/daac126)
Supplement: daac126_suppl_Supplementary_Material_1 [file daac126_suppl_supplementary_material_1.docx]

**Supplementary Material 1**

***Stage 1. Familiarisation and Raw Coding***

**Table 1.** Example of audio transcript statements and the latent and semantic coding elements. *Location anonymised

| **Audio Transcript Data** | **Semantic Coding *(Descriptive)*** | **Latent Coding *(Interpretative)*** | **Code** |
| --- | --- | --- | --- |
| “We need to be able to walk on the pavement, quite often is blocked by cars parked…cars parking on pavement smashes them up and it makes me very, very annoyed”. | The presence of cars parking on pavements stops walking and damages pavements. | Cars parking on pavement is a barrier to active ageing as the individual is “***very,*** ***very annoyed”*** by this as they “***need to be able to walk”*** but the parked cars prevent them from doing so. | Pavement blocked by parked cars. |
| “I love our tree lined streets…it’s quite leafy and pleasant here”. | Trees are aesthetically pleasing and a positive feature. | Tree lined streets are a facilitator as the individual ***loves*** the tree lined streets and it makes it more ***pleasant*** when spending time outside. | Pleasant tree lined streets. |
| “it is so bad with transport. Some people not even following the rules, they are like going zigzags. It's so scary to take your car from entering into from area 1* all the way to area 2*, it is horrendous and people are not following the proper rules”. | Drivers behaviour on the road negatively impacts the time spent outside. | Drivers and their behaviour are a barrier as the individual finds it ***scary*** to go out in their car and ***horrendous*** that other drivers don’t follow the rules. | Drivers not following rules. |

**Urban Barrier and Facilitator Themes Diagram**


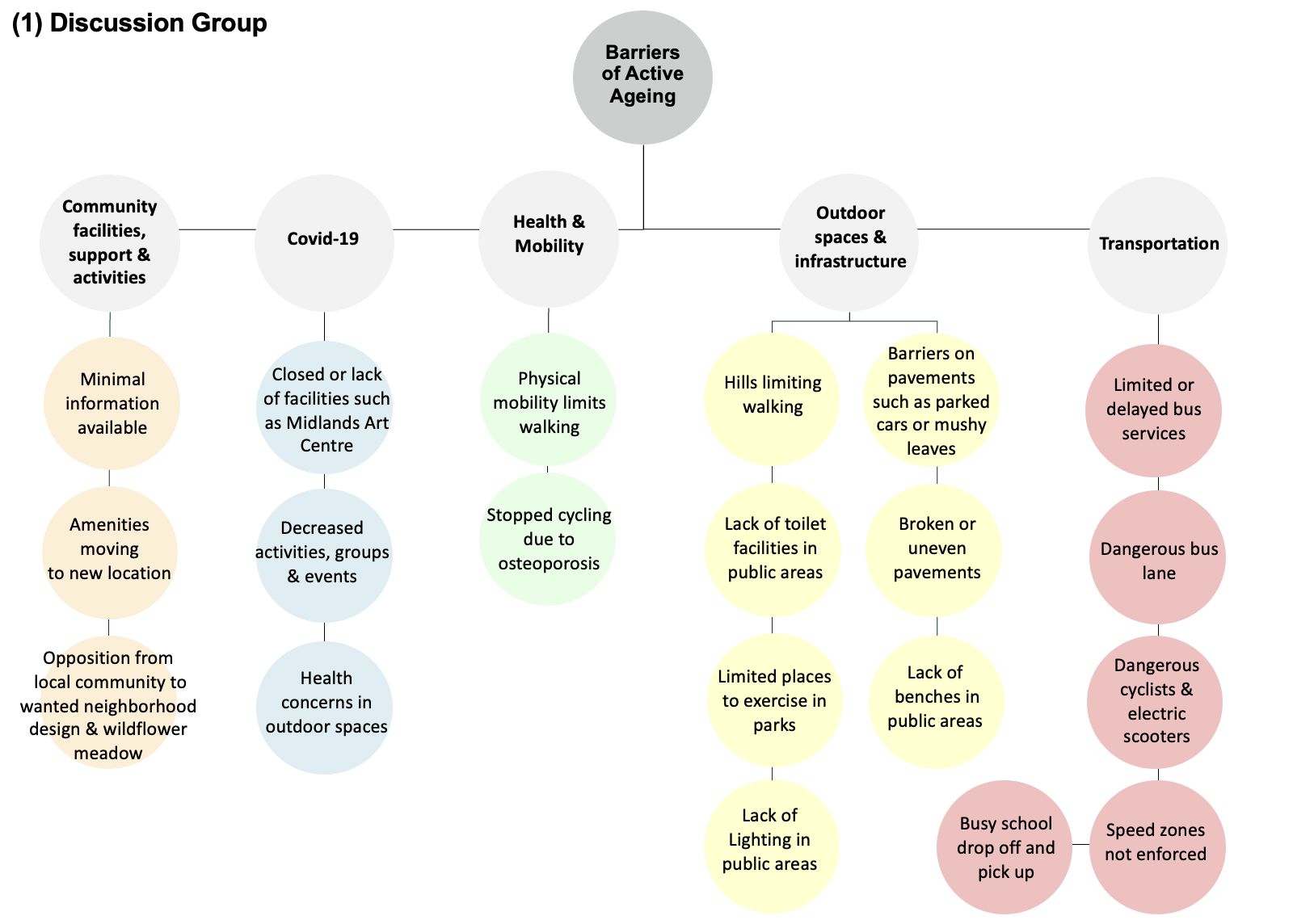


**Figure 3.** Example of barrier themes diagram shared with citizen scientists


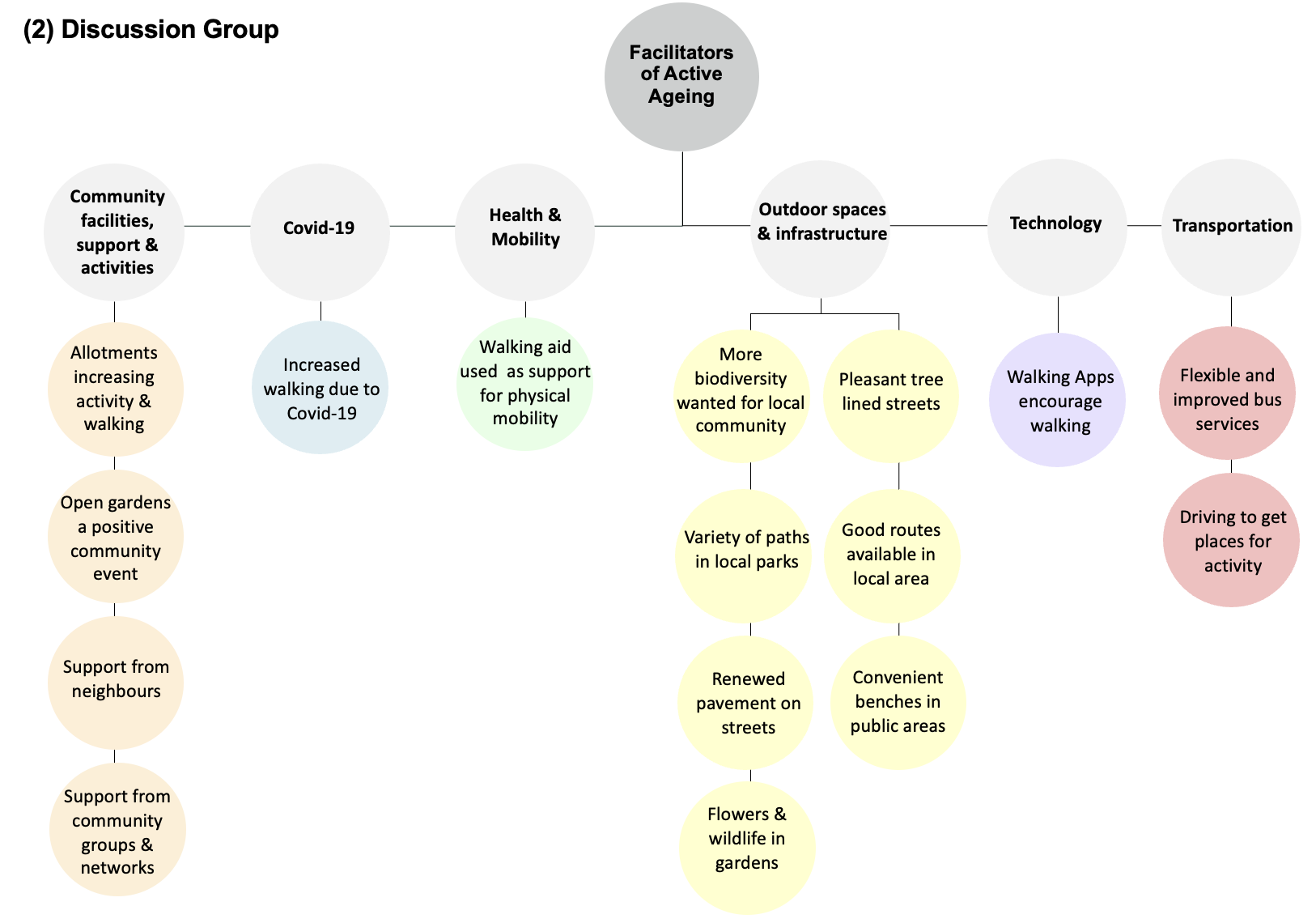


**Figure 4.** Example of facilitator themes diagram shared with citizen scientists
