# Supplementary material for: Active and healthy ageing in urban environments: laying the groundwork for solution-building through citizen science
Source: Health Promot Int. 2022 Sep 14;37(4):daac126. doi: 10.1093/heapro/daac126 (PMC9472256; doi:10.1093/heapro/daac126)
Supplement: daac126_suppl_Supplementary_Material_2 [file daac126_suppl_supplementary_material_2.docx]

**Supplementary Material 2**

**Table 1.** Common thematic trees of older adult discussion groups.

| **Main Thread** | **Main Themes** | **Subthemes** | **Codes** |
| --- | --- | --- | --- |
| Barriers of active ageing | Community facilities, support & activities | Lack of information | Minimal information available |
|  |  |  | Information unavailable |
|  |  |  | Information not communicated |
|  |  |  | Feeling cut off |
|  |  |  | Physical information unavailable |
|  |  |  | No vehicle for providing Information |
|  |  |  | Privatised services reducing Information |
|  |  |  | No advertisement of information |
|  |  |  | Minimal information for public toilets location |
|  |  |  | Information out of date |
|  |  | Lack of suitable and accessible facilities, venues & services | Doctors surgery moving to new location |
|  |  |  | Pelican crossing wanted to reduce traffic |
|  |  |  | Council services lacking |
|  |  |  | Council services only completed if there is a danger |
|  |  |  | Local access & provision to health care services needed |
|  |  |  | Health care services not good enough |
|  |  |  | Venues too expensive |
|  |  |  | No age-friendly access |
|  |  |  | Access not a priority to council |
|  |  |  | Handyman needed for support at home |
|  |  |  | Improvements too expensive |
|  |  |  | Waiting list to access allotment |
|  |  |  | Few vegetarian Indian restaurants |
|  |  | Opposition from local community to neighbourhood design | Opposition to neighbourhood design |
|  |  |  | Opposition to uncut verges |
|  |  |  | Opposition to wildflower meadow |
|  |  |  | Opposition against trees |
|  |  |  | Opposition to public art |
|  |  | Lack of age inclusive activities and adult educations | Limited age profile for activities |
|  |  |  | Difficult to engage with younger people |
|  |  |  | Need more age inclusive projects |
|  |  |  | Limiting social and physical activity |
|  |  |  | No funding available |
|  |  |  | No common place to meet up |
|  |  | Lack of funding and individuals for community groups | Not easy to find funding |
|  |  |  | Running out of funding |
|  |  |  | Small base of individuals running groups |
|  |  | Costs of activities | Costs are too high |
|  |  | No social support for activities | No one to do activities with |
|  | Covid-19 | Closed or lack of facilities | Closed art centre |
|  |  |  | Closed cafes, restaurants & shops |
|  |  |  | Unavailable facilities for drinks or rest breaks |
|  |  |  | Takeaway only |
|  |  |  | LGBT centre closed |
|  |  |  | Concern for groups not re-opening |
|  |  |  | Unavailable information for toilets |
|  |  |  | Leisure centre not running activities |
|  |  |  | Cleaning of facilities |
|  |  | Decreased activity, groups & events | Cancelled events |
|  |  |  | Limited locations for walking |
|  |  |  | Reduced cycling due to closed destinations |
|  |  |  | Boring zoom events |
|  |  |  | Not going anywhere |
|  |  |  | Not going out due to isolation |
|  |  |  | Reduced walking |
|  |  |  | Can’t attend community groups |
|  |  |  | Can’t meet friends |
|  |  |  | Reduced travelling |
|  |  |  | Missing in-person activities |
|  |  |  | Reduced exercise |
|  |  |  | Community groups stopped |
|  |  |  | Reduced time outdoors due to increase of people |
|  |  |  | Reduced activity due to health risk |
|  |  |  | Less active due to covid |
|  |  | Health concerns | Concern for safety on transport |
|  |  |  | Reduced visibility due to face masks |
|  |  |  | Concern for being close to school children |
|  |  | Reduced community & social support | Can’t attend hospital appointments |
|  |  |  | Haven’t got enough social friends |
|  |  |  | Can’t help neighbours |
|  |  |  | No face-to-face support with digital technology |
|  |  | Safety & Crime | Increase of crime due to Covid-19 |
|  |  |  | Covid-19 rules not followed |
|  |  | Second lockdown | Negative impact on health and well-being |
|  |  | Bad weather | Decreasing exercise |
|  | Health & mobility | Activity limitations due to health | Walking limitations |
|  |  |  | Osteoporosis stopped cycling |
|  |  |  | Inactive due to health condition |
|  |  |  | Personal limitations stopping exercise |
|  | Outdoor spaces & infrastructure | Barriers on pavements | Blocked by cars |
|  |  |  | Blocked by businesses |
|  |  |  | Broken and uneven pavements |
|  |  |  | Curbs causing injury |
|  |  |  | Curbs not suitable for wheelchair users |
|  |  |  | Hazardous leaves |
|  |  |  | Muddy pathways slippy |
|  |  | Common wealth games impacting outdoor spaces | Closed off footpaths |
|  |  |  | Making park muddy |
|  |  |  | Moving and rebuilding infrastructure |
|  |  | Limited facilities, activities & paths in parks | Empty park |
|  |  |  | Unavailable facilities for food or sitting |
|  |  |  | More lighting needed |
|  |  |  | Events needed |
|  |  |  | Lack of variety of paths & places to exercise |
|  |  | Cleanliness of outdoor spaces | Cleaning of streets needed |
|  |  |  | Spaces not aesthetically pleasing |
|  |  |  | Dirty environment due to litter |
|  |  |  | Fly tipping |
|  |  | Lack of suitable lighting | More lighting needed |
|  |  |  | More street lighting needed |
|  |  |  | Current street lighting unsuitable |
|  |  |  | Lighting needed to feel safe |
|  |  |  | Unsuitable lighting for health and mobility impairments |
|  |  | Lack of suitable benches & toilets | More benches needed |
|  |  |  | Unsuitable Benches |
|  |  |  | Areas poorly supplied with benches |
|  |  |  | Availability of Toilets |
|  |  |  | Toilets used for other purposes |
|  |  |  | Lack of toilet facilities in parks |
|  |  |  | Fear of no toilets |
|  |  |  | Journey planned for available toilets |
|  |  |  | Using supermarket or shopping centre toilets |
|  |  |  | Unsuitable and low standard toilets |
|  |  | Lack of care & responsibility for green spaces | No responsibility taken |
|  |  |  | Pockets not adopted |
|  |  |  | Green spaces vandalised and littered |
|  |  | Safety in outdoor & green spaces | Crime in parks |
|  |  |  | Need to feel safe |
|  |  |  | Not safe for children |
|  |  |  | Unsafe to walk alone |
|  |  | Unsuitable design of built environment | Discourages older people from going out |
|  |  |  | Design disabling older adults |
|  |  |  | Narrow steps take time |
|  |  |  | Design unsuitable and claustrophobic |
|  |  |  | Road design reducing feeling of community |
|  |  |  | Pelican crossing wanted to reduce traffic |
|  |  |  | Hills limiting active transport |
|  |  |  | Road construction not improving roads |
|  |  | City centre | Dislike for town |
|  |  |  | Feeling unsafe |
|  |  |  | Lack of things to do |
|  |  |  | Lack of Parking |
|  | Transportation | Public transportation | Dangerous bus lane |
|  |  |  | Limited or delayed bus services |
|  |  |  | Underused bus service |
|  |  |  | Not good for connecting between areas |
|  |  |  | No information for times or routes |
|  |  |  | No technology on buses |
|  |  |  | No access to public transport |
|  |  |  | Removing guards from train |
|  |  |  | Unreliable services |
|  |  | Traffic & private transportation | Dangerous cyclists |
|  |  |  | Hazardous electric scooters |
|  |  |  | Unsafe to cycle on roads |
|  |  |  | Busy school drop off and pick up |
|  |  |  | Speed zones not enforced |
|  |  |  | Drivers not following rules |
|  |  |  | Too many cars |
|  |  |  | More traffic and parking restrictions needed |
|  |  |  | Parking causing traffic |
|  |  |  | Traffic calming measures making traffic worse |
|  |  |  | Parking on pavements and Verges |
|  |  |  | Cars speeding |
|  |  |  | Dangerous electric and mobility scooters |
|  |  |  | Not enough parking |
|  |  |  | Cyclists are annoying |
|  |  | Air quality due to traffic | Air quality a health hazard |
|  |  | Cost of Driving | Driving too expensive |
|  | Technology | Digital Exclusion | Digital literacy important |
|  |  |  | Ability to use technology needed |
|  |  |  | Difficult for older people to use |
|  |  |  | Not coping with digital technology |
|  |  |  | Need to re-train |
|  |  |  | Development of technology excluding older people |
|  |  |  | Not Natural to Use |
|  |  |  | Lacking confidence with digital technology |
|  |  |  | Lack of access to technology & wi-fi |
|  |  |  | Costs add up |
|  |  | Negative multimedia | Reducing perception of safety |
|  | Crime & Safety | Feeling of crime and feeling unsafe outside | Cautious when outside |
|  |  |  | Feeling unsafe due to closed facilities |
|  |  |  | Staying home to feel safe |
|  |  |  | Young boys intimidating and aggressive |
|  |  |  | Carrying walking pole for safety |
|  |  |  | Neighbour almost got mugged |
|  |  |  | Fear of crime more destructive |
|  |  |  | Fear of teenagers |
|  |  | Dog walkers a danger | Dogs not on leads a risk |
|  |  |  | Dog walkers not controlling dogs |
|  | Winter | Darker evenings | Reduced walking |
|  |  |  | Feeling unbalanced |
|  | Ageing | Resources for older adults | Need to consider available resources |
|  |  | Becoming isolated | Reduced social connections |
|  |  |  | World becoming smaller |
|  |  | Intergenerational challenges | Divide with younger people |
|  |  |  | Difficult to engage with younger people |
|  |  |  | More distanced from younger people |
|  |  | Lack of support for retirement | Support focuses on pensions |
|  |  |  | No awareness for going out |
|  |  | Different abilities & confidence | Losing self-confidence due to frailty |
|  |  |  | Older adults varied in abilities & confidence |
|  | Ageism | Age inclusivity issues | Society needs to be more age inclusive |
|  |  |  | Need to break down age barriers |
|  |  | Negative perception & treatment of older people | Patronised due to age |
|  |  |  | Viewed negatively |
|  |  |  | Society tries to dictate older adults |
|  | | | |
| Facilitators of active ageing | Community facilities, support & activities | Allotments increasing activity | Reason for walking & activity |
|  |  |  | Reason for social and physical activity |
|  |  | Aesthetics of allotments & facilities | Aesthetically pleasing allotments |
|  |  |  | Local facilities look nice |
|  |  | Support from surrounding community | Neighbours Help with gardening |
|  |  |  | Neighbours helping and talking to neighbours |
|  |  |  | Churches providing Christmas decorations |
|  |  |  | Churches providing food and seating |
|  |  |  | Enforcing car parking regulations |
|  |  | Available local facilities & services | Cafes increasing walking |
|  |  |  | Cafes providing toilets and seating |
|  |  |  | Socialising at pubs and restaurants |
|  |  |  | Health services available |
|  |  |  | Shops & library available |
|  |  |  | Walking to shops |
|  |  |  | Good quality toilets in shops |
|  |  |  | Supermarket toilets are useful |
|  |  |  | Good access to health services and shops |
|  |  |  | Free art centre |
|  |  | Available & inclusive activities | Many arts events available |
|  |  |  | Content with available activities |
|  |  |  | Activities that are not ability led a positive |
|  |  |  | Age inclusive groups |
|  |  |  | Positive holiday events |
|  |  | Positive community groups and events | Bournville magazine |
|  |  |  | Community club member |
|  |  |  | Litter picking |
|  |  |  | Open garden events |
|  |  |  | Increased socialising and walking |
|  |  |  | Making a positive difference in local area |
|  |  |  | Online community network |
|  |  |  | Providing digital schemes for older people |
|  |  |  | Building community cohesion |
|  | Covid-19 | Increased outdoor activity during Covid-19 | Exploring local area more |
|  |  |  | Easy to walk in the summer |
|  |  |  | Increased cycling and walking |
|  |  |  | More inventive walks |
|  |  |  | Increased walking |
|  |  |  | Exercising outdoors |
|  |  | Improved community facilities & support | Facilities providing toilets |
|  |  |  | Changes to facilities imaginative and lively |
|  |  |  | Shops more comfortable with distancing |
|  |  |  | Improved health care system |
|  |  |  | Increased community cohesion |
|  |  |  | Socially distanced activities increasing community cohesion |
|  |  |  | Love and care from local community |
|  |  |  | Community Groups focused on re-engaging |
|  |  | Connecting through Digital Technology | Keeping connected through Zoom |
|  |  |  | Activities continued online |
|  |  |  | WhatsApp increasing social support |
|  | Health & mobility | Support for walking | Walking aid a positive for activities |
|  | Outdoor spaces & infrastructure | More biodiversity wanted | Leaving uncut grass for biodiversity |
|  |  | Positive aesthetics and design of outdoor and green spaces | Aesthetically pleasing gardens |
|  |  |  | Variety of design wanted |
|  |  |  | Flowers & wildlife |
|  |  |  | Private space to spend time |
|  |  |  | Aesthetically pleasing tree lined streets |
|  |  |  | Great asset to the environment |
|  |  |  | Aesthetically pleasing area |
|  |  |  | Beautiful green spaces |
|  |  |  | Single track road more pleasant |
|  |  |  | Dropped curbs are good |
|  |  |  | Improving off road cycle routes wanted |
|  |  |  | Beautiful houses & grounds |
|  |  |  | Lovely green spaces |
|  |  | Good routes, paths & pavements available | Grass paths encourage walking |
|  |  |  | Pathways encourage walking |
|  |  |  | Good variety of alleyways and snickets |
|  |  |  | Renewed pavement chosen for walking |
|  |  | Activity in outdoor spaces | Music events wanted |
|  |  |  | Walking to link up spaces |
|  |  |  | Activities available in local parks |
|  |  |  | Increased cycling and walking |
|  |  |  | Walking in parks |
|  |  |  | Walking along the river |
|  |  |  | Walking for wildlife |
|  |  |  | Easy to cycle and walk |
|  |  |  | Many places to go |
|  |  |  | Outdoor spaces feel like countryside |
|  |  | Safe outdoor spaces | Feeling safe in local area |
|  |  |  | Lights increasing safety in parks |
|  |  |  | Private estate very safe |
|  |  |  | Feeling safe to walk |
|  |  | Features & facilities convenient and improving spaces | Facilities available for all ages |
|  |  |  | Public art brightens up places |
|  |  |  | Seating outside is pleasant & lively |
|  |  |  | Benches convenient for resting |
|  |  |  | Flowers making a difference |
|  |  | Positive infrastructure design | Single track road more pleasant |
|  |  |  | Dropped curbs are good |
|  |  |  | Improving off road cycle routes wanted |
|  |  | Accessible green spaces | Green space within walking distance |
|  |  |  | Local green Space available |
|  | Technology | Walking apps encourage activity | Encourage walking |
|  |  | Digital technology providing information | Information available on google maps |
|  |  | Increased connection, support & online activity | Connecting online and through WhatsApp |
|  |  |  | Supporting neighbours through WhatsApp |
|  |  |  | Online activities increasing exercise |
|  | Transportation | Driving | Get to places for activity |
|  |  |  | Not driving encourages walking |
|  |  |  | Only driving when necessary |
|  |  | Public transportation | Flexible bus services |
|  |  |  | Works well for travel |
|  |  |  | Increasing walking |
|  |  |  | Can manage without a car |
|  |  |  | Easy to access |
|  |  |  | Travel pass a positive |
|  |  |  | Used for travelling to cities |
|  |  |  | Accessible public transport |
|  |  |  | Free pass increasing travel |
|  |  |  | Used for physical & social activities |
|  |  | Enforcing road regulations | Speed cameras slowing traffic |
|  |  |  | Enforcing parking slowing traffic |
|  | Diversity of city | Diversity of experiences and facilities | Variety of people and experiences |
|  |  |  | Variety of cultural facilities and art activities |
|  |  |  |  |
|  |  |  |  |

**Table 2.** Common thematic trees of two expert discussion groups.

| **Main Thread** | **Main Themes** | **Subthemes** | **Codes** |
| --- | --- | --- | --- |
|  | Lack of community facilities, support & activities | Lack of information | No Information for activities |
|  |  |  | No information for available facilities |
|  |  |  | Reducing community capacity & shared spaces |
|  |  | Lack of support for activities | Individuals think they are forgotten |
|  | Covid-19 | Closed or lack of facilities and services | Low attendance reduces money for venues |
|  |  |  | Closed churches |
|  |  |  | Support & services vanished |
|  |  |  | Fear of services spreading Covid-19 |
|  |  |  | Closed shops |
|  |  |  | Facilities not re-opening due to economic impact |
|  |  |  | Decreased physical & social activity |
|  |  | Deskilled individuals | Individuals deskilled due to Shielding |
|  |  | Reduced activity | Couldn’t buy a bike |
|  |  |  | Decreased socialising |
|  |  |  | Older adults not leaving home |
|  |  | Issues with digital technology | Lack of access to technology & wi-fi |
|  |  |  | Not a priority to older adults |
|  |  |  | No face-to-face support available |
|  |  |  | Digital exclusion |
|  |  |  | Priority issue due to pandemic |
|  |  | Re-engaging in activities post Covid-19 | Anxiety for re-engaging |
|  |  |  | Need to re-skill & re-enable people |
|  |  |  | Need to feel safe indoors |
|  |  |  | Worsening performance due to no training |
|  |  |  | Less outdoor activities |
|  |  |  | Not comfortable or confident leaving home |
|  |  | Reduced use of public transport | Afraid to use public transport |
|  |  |  | Limited availability of and capacity on buses |
|  |  |  | Risk of covid-19 |
|  |  |  | Reliance on social network for transport |
|  | Demographics | Gender differences impacts engagement & movement | Women take part more than men |
|  |  |  | Gender difference for moving around |
|  |  |  | Need comfortable places for older men |
|  |  | Race & ethnicity impact active ageing | Different enabling factors for active ageing |
|  | Deprivation & Poverty | Economic deprivation and poverty limiting older adults | Economic situation limits access to resources |
|  |  |  | Cost of transport limiting travel |
|  |  |  | Poverty a huge issue |
|  |  | Level of deprivation a key barrier in Birmingham | Deprivation key barrier to tackle |
|  |  |  | IMD greatest predictor of physical inactivity |
|  | Technology | Digital exclusion | Limited or no access to technology |
|  |  |  | Costs of wi-fi & devices |
|  |  |  | Reactive digital policies |
|  | Health, mobility & person-specific barriers | Long term conditions impact activity | Predictor of physical activity uptake |
|  |  | Individual capacity & capability | Confidence & belief in capacity and capability |
|  |  | Individual perception of barriers | Perception of risk |
|  | Outdoor spaces & infrastructure | Need for inclusive spaces | Lack of wheelchair accessibility |
|  |  | Perception of safety in outdoor spaces | Canals perceived as unsafe |
|  |  |  | Green spaces have a bad narrative |
|  |  | Lack of public seating | More public seating needed |
|  |  | Lack of toilet facilities | Can’t go far without a toilet |
|  |  |  | Public toilets not available anymore |
|  |  |  | People nervous when toilets are not available |
|  |  | Barriers on pavements | Blocked by cars |
|  |  |  | Unsuitable pavements for crossing |
|  |  |  | Quality of footpaths impacts walking |
|  |  | Different location experiences | Experiences based on location |
|  |  |  | Inner and outer city have different experiences |
|  |  | Lack of wayfinding in outdoor spaces | No signage for wayfinding |
|  |  |  | Need to consider wayfinding for dementia |
|  |  | Bubbled communities not mixing | Fear of visiting other communities |
|  |  |  | Not mixing with other communities |
|  |  | Varied accessibility to activities & green spaces | Varied access to green spaces |
|  |  |  | Varied access to activities & opportunity to participate |
|  |  | Size of Birmingham | Takes time & money to navigate |
|  |  | Unsuitable infrastructure design | Infrastructure reduces safety |
|  |  |  | Unsuitable crossing facilities |
|  |  |  | Infrastructure designed for cars instead of people |
|  |  |  | Reaching local amenities difficult due to lack of crossing facilities |
|  |  |  | Road design splitting up communities |
|  | Ageism | Negative perception & attitudes of older adults | Negative attitude towards older people |
|  |  |  | Not respected or treated well |
|  |  |  | Not viewed as valid contributors |
|  |  | View of ‘older adults’ as a homogeneous group | Different needs & circumstances for different age groups |
|  |  |  | Different travel & mobility Needs |
|  |  |  | Heterogeneity and intersectionality |
|  |  |  | Term ‘Older Adult’ needs more understanding |
|  | Transport | Traffic & Private transportation | Transport is a wicked issue |
|  |  |  | Speed of traffic |
|  |  | Public Transportation | Transport is a wicked issue |
|  |  |  | Unsuitable bus stops |
|  |  |  | Perceived as awful & not good |
|  |  |  | Perceived as unsafe & dangerous |
|  |  |  | Not good at connecting across neighbourhoods |
|  |  |  | Cost of public transportation |
|  |  |  | Lack of use from older adults |
|  |  |  | Shuttle bus cut due to funding |
|  |  |  | Not connected radially |
|  | Winter | Bad weather | Fear of slipping on snow |
|  |  |  | Reduced activity in bad weather |
|  | Planning & design of Birmingham | Planning & design issues | Planning lacks specificity & direction |
|  |  |  | Gap between policy & what’s delivered |
|  |  |  | Caveat for council to do what they want |
|  |  |  | Planning documents are generic & open to interpretation |
|  |  |  | No communication & cohesion between ward plans |
|  |  |  | Lack of support & engagement for ward plans |
|  | Safety | Barrier for mobility | Safety a mobility barrier for older adults |
|  | | | |
| Facilitators of active ageing | Approaches to increasing engagement and participation | Gamification for increasing engagement and participation | Competitive element for greater uptake |
|  |  |  | Fun games for increasing activity |
|  |  |  | Extrinsic value & rewards |
|  |  | Age inclusive activities | Not identifying as ‘older adults’ |
|  |  |  | Self-worth from joining and contributing |
|  |  | Local neighbourhood approach | Keeping individuals connected & active |
|  |  |  | Cheaper than other activities |
|  |  |  | Using local assets for activities |
|  |  |  | Keeping it local |
|  |  | Participant lead | Doing something for others |
|  |  |  | Leading & running activities |
|  |  |  | More meaningful to the Individual |
|  | Community facilities, support & activities | Comfortable & safe facilities | More comfortable in churches & community facilities |
|  |  |  | Feeling safe in pubs |
|  |  | Facilities encourage activity | Brand new leisure centre |
|  |  |  | Opportunities to take part |
|  |  |  | Facilities offering drinks |
|  |  | Community assets and activities available | 1400 assets & activities available |
|  |  |  | Self-sustaining community activities |
|  |  |  | Music activity encourages physical activity |
|  |  |  | Activities happening across the city |
|  |  | Support from community schemes | Online classes increasing confidence |
|  |  |  | Working with digital technology |
|  |  |  | Improving infrastructure for active travel |
|  |  |  | Improving local parks |
|  |  |  | Supporting community assets & needs |
|  |  |  | Improving engagement and accessibility in open spaces |
|  |  |  | Removing barriers in open spaces |
|  |  |  | Older adult focused schemes |
|  |  |  | Providing bikes for cycling initiative |
|  |  | Social & companionship support | Social element bringing people together |
|  |  |  | Building social connections through activities |
|  |  |  | Sociability just as important as physical activity |
|  |  |  | Engaging in activities for companionship |
|  |  | Community & stealth activities available | Self-sustaining community activities |
|  |  |  | Music activity encourages physical activity |
|  |  |  | Activities happening across the city |
|  | Covid-19 | Digital technology providing support | Community support through technology |
|  |  |  | Individuals becoming more digitally active |
|  |  |  | Online activities increasing confidence & support |
|  |  |  | Support & services moved online |
|  |  |  | Making digital technology accessible |
|  |  | Increased outdoor activity due to Covid-19 | Exploring neighbourhoods |
|  |  |  | Increased gardening |
|  |  |  | Increased walking and cycling |
|  |  |  | Staying active through online fitness and dance |
|  |  | Increased local community & social support | Deepened friendships through Covid-19 |
|  |  |  | Increased community cohesion & support |
|  |  |  | Support from neighbours with transport |
|  |  |  | Support from volunteers to keep connected |
|  |  |  | Individuals with mental health conditions reconnecting |
|  |  |  | Positive re-connections with others |
|  |  | Adapting services post covid-19 instead of restarting | Adapt current services instead of starting again |
|  |  | Re-engaging face-to-face activities, support & services post-covid-19 | Picking up Services, volunteering & support again |
|  |  |  | Making activities safe |
|  |  |  | Need for more cooking classes |
|  |  |  | Enabling local neighbourhood activities to continue |
|  |  | Keeping people connected post-covid-19 | Findings ways to keep people connected |
|  |  |  | Using socially distanced activities |
|  | Outdoor spaces & infrastructure | Activities in outdoor spaces | Available parks & open spaces |
|  |  |  | Community assets running activities |
|  |  | Positive aesthetics and well-kept outdoor spaces | Nice green space |
|  |  |  | Well-kept canal tow paths encourages activity & feeling Safe |
|  |  | Inclusive changes to design & infrastructure | Design changes interesting & engaging |
|  |  |  | Making facilities such as toilets and seating accessible positive for all age groups |
|  |  | Narrative of outdoor spaces | Need to change narrative of green spaces |
|  |  | Wayfinding & signs | Improving signage increases exploration & wayfinding |
|  | Transportation | Low traffic neighbourhood | Encourages physical activity |
|  |  | Public transportation | Perceived as good |
|  |  |  | Perceived as safe |
|  |  |  | Frequent bus service |
|  |  |  | Good for going to town |
|  |  | Social network & private transport | Driving or social network used for journeys |
